# First report of *Amblyomma sculptum* (*Amblyomma cajennense* complex) in a Brazilian state classified as a silent area for human rickettsiosis

**DOI:** 10.14202/vetworld.2023.2200-2204

**Published:** 2023-11-01

**Authors:** Bruna Costa da Gama, Thiago Fernandes Martins, Marcelo Bahia Labruna, Rafael Felipe da Costa Vieira, Jonatas Campos de Almeida

**Affiliations:** 1 Centro de Engenharia e Ciências Agrárias, Universidade Federal de Alagoas, Viçosa, Alagoas, Brazil; 2 Departamento de Medicina Veterinária Preventiva e Saúde Animal, Faculdade de Medicina Veterinária e Zootecnia, Universidade de São Paulo, São Paulo, São Paulo, Brazil; 3 Instituto Pasteur, Área Técnica de Doenças Vinculadas a Vetores e Hospedeiros Intermediários, Secretaria de Estado da Saúde de São Paulo, São Paulo, Brazil; 4 Department of Public Health Sciences, College of Health and Human Services, The University of North Carolina at Charlotte, North Carolina, USA; 5 Center for Computational Intelligence to Predict Health and Environmental Risks, The University of North Carolina at Charlotte, North Carolina, USA

**Keywords:** *Amblyomma sculptum*, public health, silent area

## Abstract

**Background and Aim:**

Studies on ticks of public health concern in equine husbandry are scarce in the Northeastern region of Brazil. This study aimed to investigate the presence of ticks on horses in the State of Alagoas, which is classified as a silent area for human rickettsiosis.

**Materials and Methods:**

Ticks infesting horses were collected using anatomical tweezers or a commercial hook and kept in ethanol-labeled tubes for taxonomic identification.

**Results:**

A total of 2,238 ticks were found. Ticks were identified as 2,215 (98.89%, 95% CI: 98.41–99.28) *Dermacentor nitens*, 19 (0.98%, 95% CI: 0.05–1.38) *Amblyomma sculptum*, and 4 (0.18%; 95% CI: 0.007–0.46) *Rhipicephalus microplus*.

**Conclusion:**

This is the first study to report *A. sculptum* and *D. nitens* in the State of Alagoas. The presence of *A. sculptum* should draw the attention of public health managers once Alagoas State is considered a silent area for rickettsial diseases, which means the absence of local surveillance programs for these pathogens.

## Introduction

*Amblyomma sculptum* Berlese, 1888 [1], is a hard tick that belongs to the *Amblyomma cajennense* species complex. It is widely distributed in the Brazilian territory, mainly in the Cerrado and Pantanal Biomes and deforested areas of the Atlantic rainforest [2–4]. *Amblyomma sculptum* is the most common human-biting tick in Brazil [4–6] and is the primary vector of *Rickettsia rickettsii*, the etiological agent of Brazilian spotted fever, a re-emerging zoonosis with high lethality [7]. Capybaras (*Hydrochoerus hydrochaeris*), tapirs (*Tapirus terrestris*), and horses are the preferred hosts of *A. sculptum* [4].

The daily use of equines in activities such as traction, work, sports, and treating diseases (equine therapy) has created an interface with humans, facilitating the sharing of zoonotic parasitic diseases. In view of this, the equine capacity as a sentinel animal for some zoonoses is an aspect that should be better monitored and applied as a tool in the one health approach [8, 9]. *Amblyomma sculptum* is a significant threat to the creation and maintenance of horses, impacting their health and welfare once it is suspected to act as a vector of the piroplasm *Theileria equi* [10]. Despite this, studies on ticks in equine husbandry are even more scarce in the Northeastern Region of Brazil, with few studies evaluating the occurrence of hard ticks in other domestic and wild animals in the states surrounding Alagoas, such as Bahia and Pernambuco [4, 11]. In the Brazilian State of Alagoas, there is a lack of information about ticks, which are a public health concern parasitizing horses. Moreover, Alagoas is considered to be a silent area or an area without a human case of rickettsial disease [12]. A ”silent” area has or is likely to have unreported cases of a disease. In this scenario, it is extremely difficult to establish prevention and control measures for rickettsial diseases because there are no data on the prevalence or incidence to define which areas are most at risk and require greater epidemiological and acarology surveillance. Despite a possible disease outbreak, local public health authorities will face greater difficulty in controlling the situation [13].

This study aimed to investigate the presence of ticks on horses in the State of Alagoas, with an emphasis on the search for *A. sculptum*, a tick of major public health importance in Brazil.

## Materials and Methods

### Ethical approval

The use of animals in this study followed ethical and animal welfare principles. The Ethics Committee for Animal Use approved and certified this study at the Federal University of Alagoas (protocol number 22/2021).

### Study period and location

**Figure-1:**
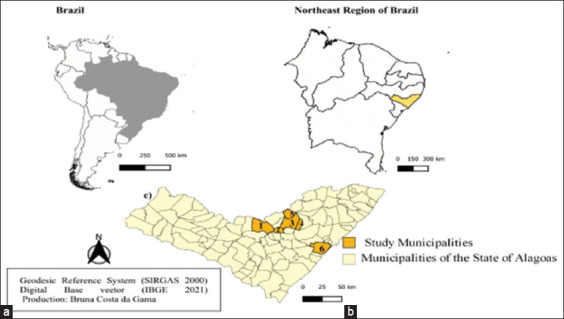
Geographical locations of horse farms used in the present study. (a) South America–Brazil; (b) Northeastern Region of Brazil; (c) Horse farms used in the present study. State of Alagoas, Brazil, 2022 (1=Palmeiras dos Índios; 2=Mar Vermelho; 3=Viçosa; 4=Cajueiro; 5=Chã Preta; 6=Marechal Deodoro) [Source: The map was generated using QGIS version 2.23.3].

The study was conducted from May 2022 to February 2023. The State of Alagoas (Figure-1) has an area of 27,731 km^2^ [14], a tropical climate (Am Köppen: Am), irregular rainfall (800–1200 mm) throughout the year, and an average temperature between 22°C and 29°C.

To improve the odds of finding ticks of public health importance, all farms were chosen based on the previous occurrence of tick-borne diseases in dogs of the property. Thus, six properties that met this requirement were selected for this investigation. The animals belonged to the coastal regions and the inland regions.

### Animal and body samples

Horses were chosen by convenience, mainly to avoid stress. Each animal was examined for 10 min and ticks were collected in 70% ethanol [15, 16]. Ticks infesting horses were collected using anatomical tweezers or a commercial hook (O’Tom/Tick Twister^®^, Lavancia, FRA) and kept in ethanol-labeled tubes for taxonomic identification [3, 4, 10, 17]. Sampling was performed from May to August 2022.

## Results and Discussion

**Figure-2:**
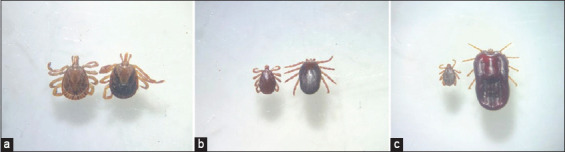
Ticks found on horses in Alagoas. (a) Male and female of *Amblyomma sculptum*; (b) Male and female of *Dermacentor nitens*; (c) Male and female of *Rhipicephalus microplus*.

A total of 2,238 ticks were collected from 305 horses, yielding an overall occurrence of 80.05% (305/381). Ticks were identified as 2,215 (98.89%, 95% CI: 98.41–99.28) *Dermacentor nitens*, 19 (0.98%, 95% CI: 0.05–1.38) *A. sculptum*, and 4 (0.18%; 95% CI: 0.007–0.46) *Rhipicephalus microplus* (Figure-2). Most of the ticks were of the adult stage (1,369 specimens; 61.14%; 907 females and 462 males), followed by larvae (444; 19.83%) and nymphs (426; 19.03%). The occurrence values per tick species were 77.4% (285/381) for *D. nitens*, 2.4% (9/381) for *A. sculptum*, and 0.3% (1/381) for *R. microplus* (Table-1).

**Table-1:** Ticks infesting horses in the state of Alagoas, Brazil, during May–August 2022.

Municipality	No. of sampled horses	No. of infested horses (% occurrence) according to tick species				Overall MI*	Overall MA*

		*Dermacentor nitens*	*Amblyomma sculptum*	*Rhipicephalus microplus*	Total		
Chã Preta	30	10 (33.3)	0	1 (3.3)	11 (36.7)	5.0	1.8
Cajueiro	120	98 (81.7)	8 (6.6)	0	106 (88.3)	5.6	4.9
Mar Vermelho	120	109 (90.1)	0	0	109 (90.8)	8.3	7.6
Viçosa	20	11 (55.0)	0	0	11(55.0)	10.4	5.7
P. dos Índios	20	4 (20.0)	0	0	4 (20.0)	12.0	2.4
Mal. Deodoro	71	63 (88.7)	1 (1.4)	0	64 (90.1)	6.8	6.1
Total	381	295 (77.4)	9 (2.4)	1 (0.3)	305 (80.0)	7.3	5.9

* MI (mean intensity)=No. of collected ticks/No. of infested horses; MA (mean abundance)=No. of collected ticks/No. of examined horses

Thus, although the three identified tick species (*D. nitens*, *A. sculptum*, and *R. microplus*) have been commonly found parasitizing horses in different regions of Brazil [18–23], to the authors’ knowledge, this is the first report of *A. sculptum* and *D. nitens* ticks identified in the State of Alagoas. *Rhipicephalus microplus* has already been previously reported by Aragão *et al*. [24] in Alagoas. The new findings of these ticks have been deposited in the tick collection “Coleção Nacional de Carrapatos Danilo Gonçalves Saraiva” (CNC) under accession numbers CNC-4578, 4579, and 4580.

*Amblyomma sculptum* was found in two municipalities: Cajueiro (09° 23′ 48″ S 36° 09′ 13″ W) and Marechal Deodoro (09° 42′ 36″ S 35° 53′ 42″ W), which are approximately 65 km from each other. Both cities have a dry summer and rainy winter, with temperatures ranging from 22°C to 29°C and an estimated altitude of 102 m and 31 m, respectively [25]. It is important to emphasize that Alagoas borders Pernambuco state, which reported an autochthonous case of rickettsial disease [26].

The travel distance from Cajueiro to Maceió (capital city) is 72 km. All horses (n = 120) were evaluated on the property, and 14 specimens of *A. sculptum* were found (six females, three males, and five nymphs) and 4 larvae of *Amblyomma* spp. The animals were raised in an extensive production system, mainly for work, and the mares were intended for reproductive purposes. Capybaras, marsupials, and synanthropic rodents were reported in environments near the horses.

The travel distance from Marechal Deodoro to Maceió is 29 km. All horses (n = 71) were evaluated on the property, and only one female specimen of *A. sculptum* was found. The animals were raised in an extensive production system, but mainly for sporting competitions and work, and the mares were intended for reproductive purposes. The presence of capybaras was described in environments close to the horses.

The present study was conducted only during the rainy season (fall/winter), and it was not possible to discuss aspects of the distribution of *A. sculptum* ticks due to seasonality. However, the higher number of adult ticks found in this study agrees with other authors [27], who reported a higher prevalence of adult ticks during the rainy season in other Brazilian regions. It is evident the importance of seasonality studies of *A. sculptum* in the Northeastern region of Brazil that evaluates a longer period, including the dry and rainy seasons.

The presence of capybaras and wild animals sharing environments with horses was described in both properties. Capybaras are considered primary hosts of *A. sculptum* and amplifiers of *R. rickettsii* [28, 29]. The use of equines for sports and daily work results in a close interface with humans, favoring the life cycle of three-host ticks and increasing the odds of the zoonotic transmission of tick-borne pathogens, especially rickettsial diseases. The ecological imbalance caused by human activities stimulates the approach of wildlife and parasites to the domestic environment [30, 31].

## Conclusion

This study reported *A. sculptum* and *D. nitens* parasitizing horses in the State of Alagoas. Further studies are necessary to determine the role of these ticks as vectors of zoonotic pathogens in Northeastern Brazil.

## Authors’ Contributions

BCG, TFM, MBL, RFCV, and JCA: Study design and conception, drafted the manuscript, and performed data analysis. BCG: Literature review, drafted and revised the manuscript, and sample collection. All authors have read, reviewed, and approved the final manuscript.
